# Polymyxin B-Induced Skin Hyperpigmentation

**DOI:** 10.1155/2020/6461329

**Published:** 2020-09-18

**Authors:** Xueke Wen, Chunliu Luo, Weitao Lyu

**Affiliations:** ^1^The First Clinical Medical College of Jinan University, No. 601 Huangpu Avenue West, Guangzhou City 510630, Guangdong Province, China; ^2^The First Affiliated Hospital of Jinan University, No. 613 Huangpu Avenue West, Guangzhou City 510630, Guangdong Province, China

## Abstract

Polymyxin B-induced skin hyperpigmentation is a rare adverse drug reaction (ADR). In this report, we present the case of a patient who underwent an abscess resection with right epididymitis, in which a multidrug-resistant *Klebsiella pneumoniae* infection (KPI) is formed. The patient was treated with polymyxin B and subsequently developed skin hyperpigmentation and desquamation. The desquamation improved and the pigmentation gradually returned to normal after sixty days after the withdrawal of polymyxin B.

## 1. Introduction

Polymyxin B is a polypeptide antibiotic that has bactericidal activity against aerobic Gram-negative bacteria [1, 2]. Nephrotoxicity and neurotoxicity are the most common adverse drug reactions (ADRs) of intravenous polymyxin B therapy [3]. Furthermore, cases of polymyxin B-induced skin hyperpigmentation have not been reported in the recent literature. Currently, the pathogenesis of polymyxin B-induced skin hyperpigmentation is controversial and remains unknown. Skin hyperpigmentation not only affects the patient's appearance but also affects the patient's quality of life and may be associated with psychological problems. We present the case of severe skin hyperpigmentation following the administration of polymyxin B. This study was conducted according to the principles of the Declaration of Helsinki and approved by the ethics review committee of our institution. The patient provided informed consent for the publication of this report.

## 2. Case Presentation

**Figure 1:**
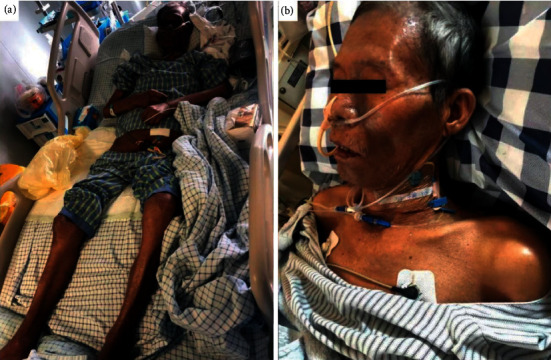
Photograph of the patient on (a) Day 17 of use polymyxin B and (b) Day 60 after the withdrawal of polymyxin B. A 68-year-old man received polymyxin B therapy for a *Klebsiella pneumoniae* blood infection. With treatment, the patient had progressive hyperpigmentation (a) that significantly faded after the polymyxin B was withdrawn (b).

A 68-year-old man presented with acute swelling of his right scrotum with obvious pain. His body temperature was 38.4°C. After physical and radiological examinations, the patient was diagnosed with right epididymitis with an abscess. He was admitted to the urological surgery department for antibacterial therapy for two weeks. Subsequently, he underwent a resection of the right epididymis and abscess. However, 24 hours after surgery, he experienced septic shock and was transferred to the intensive care unit for further treatment. A blood culture suggested a *Klebsiella pneumoniae* infection (KPI). The patient was treated with cefepime, imipenem, piperacillin-tazobactam, and tigecycline, but his symptoms did not improve. Subsequently, he was confirmed to have multidrug-resistant KPI. Polymyxin B (500,000 units, intravenous, *q* 12 hours) was administered with meropenem (2 g, intravenous, *q* 8 hours) and tigecycline (100 mg, intravenous, *q* 12 hours), and his symptoms improved. On the 8th day of polymyxin B therapy, the patient developed a red, scattered, dotted, pruritic rash on his trunk and limbs. On the 14th day of polymyxin B therapy, the rash subsided and the pruritus slightly improved; however, the patient's face and neck changed from a normal yellow color to a slightly black color. Despite this adverse event, polymyxin B therapy was continued for a total of 17 days due to its effectiveness against the multidrug-resistant KPI. Seven days after the withdrawal of polymyxin B, the color of the patient's face and neck was completely black (Figure 1(a)). The patient's scalp and feet underwent desquamation, which lasted for 10 days. Cod liver oil ointment was administered three times a day to lubricate and protect the newly grown epidermis. The patient was advised to let the skin layer fall off naturally and not to peel it in order to prevent a skin infection.

One nurse was assigned to care for the patient and record the changes in hyperpigmentation daily. The patient and his family were counseled regarding the psychological effects of the hyperpigmentation. Twenty-four days after the withdrawal of polymyxin B, the skin around the patient's eyes and nose began to fade significantly, followed gradually by the whole face and neck. The desquamation of the patient's feet improved. Sixty days after the withdrawal of polymyxin B, the hyperpigmentation of the patient's face and neck skin was almost entirely resolved (Figure 1(b)).

## 3. Discussion

Polymyxin was first obtained from *Bacillus* bacteria in the late 1940s and has been used worldwide as the last therapeutic bacterium against carbapenem-resistant Gram-negative bacteria since the 1960s [4, 5]. The ADRs of intravenous polymyxin B include allergic reactions, dyspnea, tachycardia, eosinophilia, fever, nephrotoxicity, and neurotoxicity [3]. This report suggests that skin hyperpigmentation is also an ADR of intravenous polymyxin B.

Dai et al. have described the roles of the mitochondrial, death receptor, endoplasmic reticulum, and MAPK pathways in colistin-induced nephrotoxicity [6]. The MAPK pathway can also regulate the release of histamine by basophils, mast cells, and neurons in skin tissues, which can activate the inflammatory reaction by acting on four receptors [7]. Yoshida et al. reported that histamine activates the H2 receptors of melanocytes, upregulating the activities of both tyrosinase and protein kinase A [8]. Protein kinase A plays a key role in melanogenesis [8]. Therefore, polymyxin B may induce the release of histamine and the synthesis of melanin, leading to skin hyperpigmentation [9]. The mechanism of skin hyperpigmentation induced by polymyxin B is likely multifactorial. It has been reported that Langerhans cell proliferation and the overexpression of IL-6 in the skin may be caused by the inflammatory response following the use of polymyxin B [9]. As polymyxin B causes the release of histamine, which is regulated by the MAPK pathway that produces melanin, skin darkening may be associated with histamines, skin hyperpigmentation, and inflammatory processes in response to intravenous polymyxin B treatment [6]. Histamine release caused by polymyxin B may play a role in melanocyte activation and skin hyperpigmentation [5, 10]. Most patients with skin hyperpigmentation after the administration of polymyxin B were administered this antibiotic due to a multidrug-resistant infection. Therefore, the sequential administration of more than one antibiotic may also play a role in skin hyperpigmentation [11, 12].

The patient in this case report developed skin changes eight days after the initiation of polymyxin B therapy, and the face and neck skin was completely black on the seventh day after the drug was withdrawn. Furthermore, the patient's skin recovered slowly after the cessation of the administration of polymyxin B. Polymyxin B-induced skin hyperpigmentation has been found to be related to MDR [6]. More research is necessary to determine the exact causes of skin hyperpigmentation induced by polymyxin B.

Skin hyperpigmentation induced by polymyxin B not only affects the appearance but also stresses the patient mentally. A patient's mood may be greatly affected by skin hyperpigmentation. The treatment plan for hyperpigmentation should include counseling and emotional support by a dedicated, experienced professional throughout the patient's recovery.

## Data Availability

The data used to support the findings of this study are available from the corresponding author upon request.
